# PKC Dependent p14ARF Phosphorylation on Threonine 8 Drives Cell Proliferation

**DOI:** 10.1038/s41598-018-25496-4

**Published:** 2018-05-04

**Authors:** Rosa Fontana, Daniela Guidone, Felicia Sangermano, Viola Calabrò, Alessandra Pollice, Girolama La Mantia, Maria Vivo

**Affiliations:** Department of Biology, Università degli Studi di Napoli “Federico II”, Napoli, Italy

## Abstract

ARF role as tumor suppressor has been challenged in the last years by several findings of different groups ultimately showing that its functions can be strictly context dependent. We previously showed that ARF loss in HeLa cells induces spreading defects, evident as rounded morphology of depleted cells, accompanied by a decrease of phosphorylated Focal Adhesion Kinase (FAK) protein levels and anoikis. These data, together with previous finding that a PKC dependent signalling pathway can lead to ARF stabilization, led us to the hypothesis that ARF functions in cell proliferation might be regulated by phosphorylation. In line with this, we show here that upon spreading ARF is induced through PKC activation. A constitutive-phosphorylated ARF mutant on the conserved threonine 8 (T8D) is able to mediate both cell spreading and FAK activation. Finally, ARF-T8D expression confers growth advantage to cells thus leading to the intriguing hypothesis that ARF phosphorylation could be a mechanism through which pro-proliferative or anti proliferative signals could be transduced inside the cells in both physiological and pathological conditions.

## Introduction

The p14ARF protein, encoded by the INK4a/ARF locus, was initially described as a tumor suppressor that, in response to different oncogenic stimuli, by protecting p53 from proteasome mediated degradation, initiates a cell pathway leading to cell cycle block and/or apoptosis^1,2^. Further studies indicated that, apart from p53, ARF functionally interacts with a number of factors, thus mediating cellular response also through p53-independent activities^3^. ARF has unexpectedly been found over expressed or stabilized in several types of cancers^4–6^, to be involved in autophagy^7–9^ and, recently, to have a role in protecting human melanocytes from free radicals arising upon mitochondrial dysfunction^10^. In addition to this, it also appears to play a role during development^11–13^. These observations led to the conclusion that, somehow, ARF role within the cell can be highly pleomorphic or context-dependent, ranging from halting uncontrolled cell proliferation in some cases to favour cancer growth in others. We recently demonstrated that ARF plays an unexpected role in the cytoplasm in the organization of the cytoskeleton. During cell adhesion, ARF accumulates at sites of polymerized actin such as focal adhesions, where it co-localizes with and induces activation of the Focal Adhesion Kinase (FAK). Interestingly, this mechanism appears to be conserved in mouse. By aiding cytoskeleton assembly during spreading, ARF protects cells from anoikis blocking DAPK (Death Associated Protein Kinase) dependent apoptosis^14^.

We previously demonstrated that ARF is regulated through the activation of PKC pathway in both cancer and transformed cell lines^15^. The involvement of phosphorylation in controlling ARF activities has been suggested by different experimental approaches^16–19^. *In vitro* kinase assay shows that three PKC consensus sites identified in silico within ARF sequence are specifically phosphorylated by PKC. In addition, we show that the protein is phosphorylated *in vivo*^15^. Mimicking the un-phosphorylatable status of the protein on Threonine 8 (T8A mutant), confers instability to the protein while not affecting its ability to restrain cell proliferation. Conversely, the T8D ARF mutant, that corresponds to the constitutive phosphorylation status of the protein, accumulates in the cytoplasm and is less efficient than the wt in halting cell proliferation. These data led to the hypothesis that ARF function might be regulated by phosphorylation on this conserved residue. PKC plays important role in a number of cell functions^20^. Among these, it has been shown that it is involved in the regulation of cell morphology^21^ through the phosphorylation of a high number of proteins involved in cell migration and in the generation of focal adhesion^22,23^.

On the basis of this evidence, we sought to investigate if ARF role in cell spreading and its functional relation with FAK could be regulated by PKC activity. Here we show that during cytoskeleton remodelling induced by cell spreading, ARF protein levels increase in the cytoplasm through a PKC dependent mechanism. Mimicking the phosphorylation status of the protein is sufficient to drive its localization in the cytoplasm and to rescue spreading defect as well as FAK phosphorylation of ARF silencing in HeLa cells, thus resulting in an increased proliferative ability. Taken together these data indicate that PKC activation can prime ARF involvement in cell spreading leading to increased FAK activation and cell proliferation.

## Results

### Threonine to Aspartic mutation in Threonine 8 is sufficient to affect ARF localization

**Figure 1:**
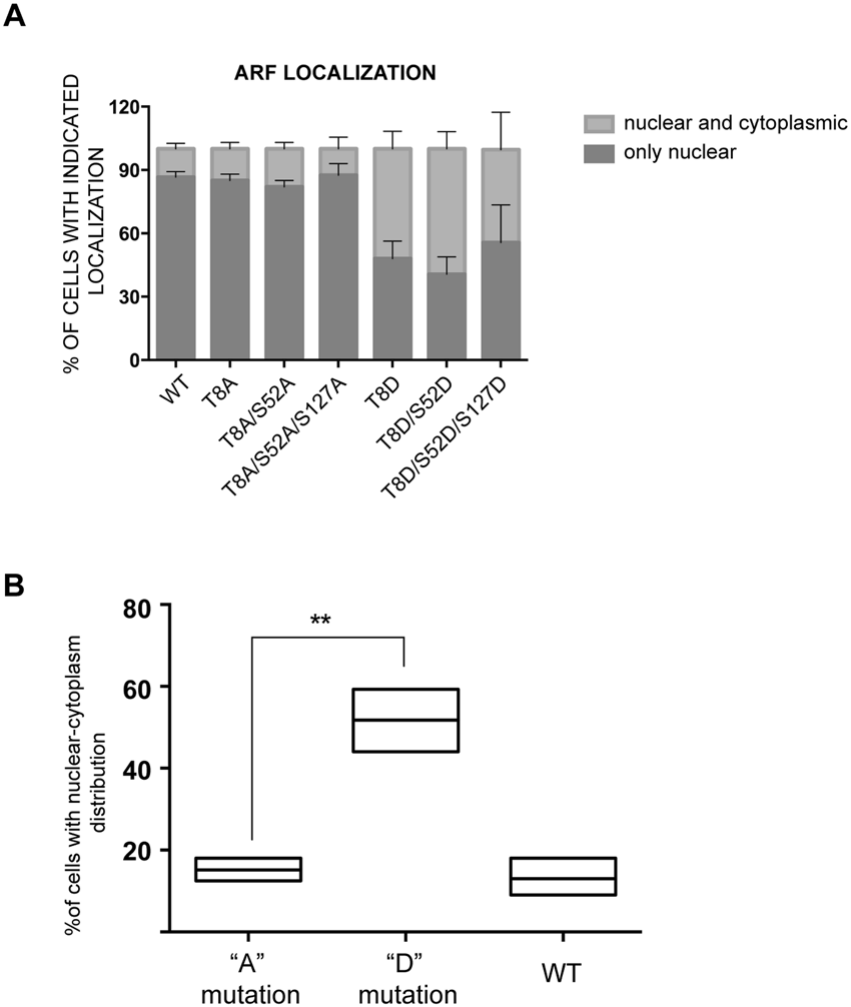
Threonine 8 mutation affects ARF localization. (**A**) percentage of transfected cells showing nuclear and nuclear-cytoplasmic localization pattern + SD, n = 3. (**B**) nucleo-cytoplasmic localization of mutants of the A series vs D series. Analysis of variance by two-tailed paired t-test, **P = 0.0060.

The threonine 8, lying in the most conserved region of the protein, is also highly conserved within ARF protein sequence of different species. To analyse the relation between this site and the other PKC consensus sites (serine residues in position 52 and 127^15^), we constructed double (T8-S52) and triple (T8-S52-S127) mutants in which each single potential PKC site was replaced either with an alanine (“A” serie), that cannot be phosphorylated, or with an aspartic acid (“D”), that mimics the phosphorylation status of the protein. ARF protein displays various degree of accumulation in nucleoli and/or scattered throughout the nucleoplasm^24,25^. We then tested if the inserted mutations could affect ARF subcellular localization evaluating subcellular localization of tagged WT and mutant ARF proteins transfected in U2OS cells by IF with anti Hys antibody. For each mutant, we counted the number of transfected cells displaying nuclear (Fig. S1, nucleolar + diffuse nuclear, left and middle panel) and nucleo-cytoplasmic localization (Fig. S1 right panel) and these data were reported in a graph of Fig. 1A. Immunofluorescence experiments showed that both double and triple mutations mimicking the un-phosphorylatable status of the protein, display a localization pattern similar to WT and T8A mutant performed as control (Figs 1A and S1). In contrast, the double and triple mutants of the “D” series localize both in the nucleus and in the cytoplasm in almost 50% of transfected cells (Fig. 1A), as previously reported for the T8D mutant^15^. These results suggest that Thr8 mutation alone is sufficient to determine ARF localization. This allowed us to statistically analyse the role of T to A mutations vs T to D mutations. Comparing the percentages of nucleo-cytoplasmic localization of mutants of the A series with those of the D series, we obtained the plot shown in Fig. 1B. We could observe how the localization of un-phosphorylatable ARF proteins (as well as of the WT) is significantly different from that of the “D” mutants. Collectively these results suggest that mimicking phosphorylation on threonine 8 alone is the signal sufficient to induce ARF accumulation in the cytoplasm.

### ARF is induced in the cytoplasm during cytoskeleton reorganization through PKC activation

**Figure 2:**
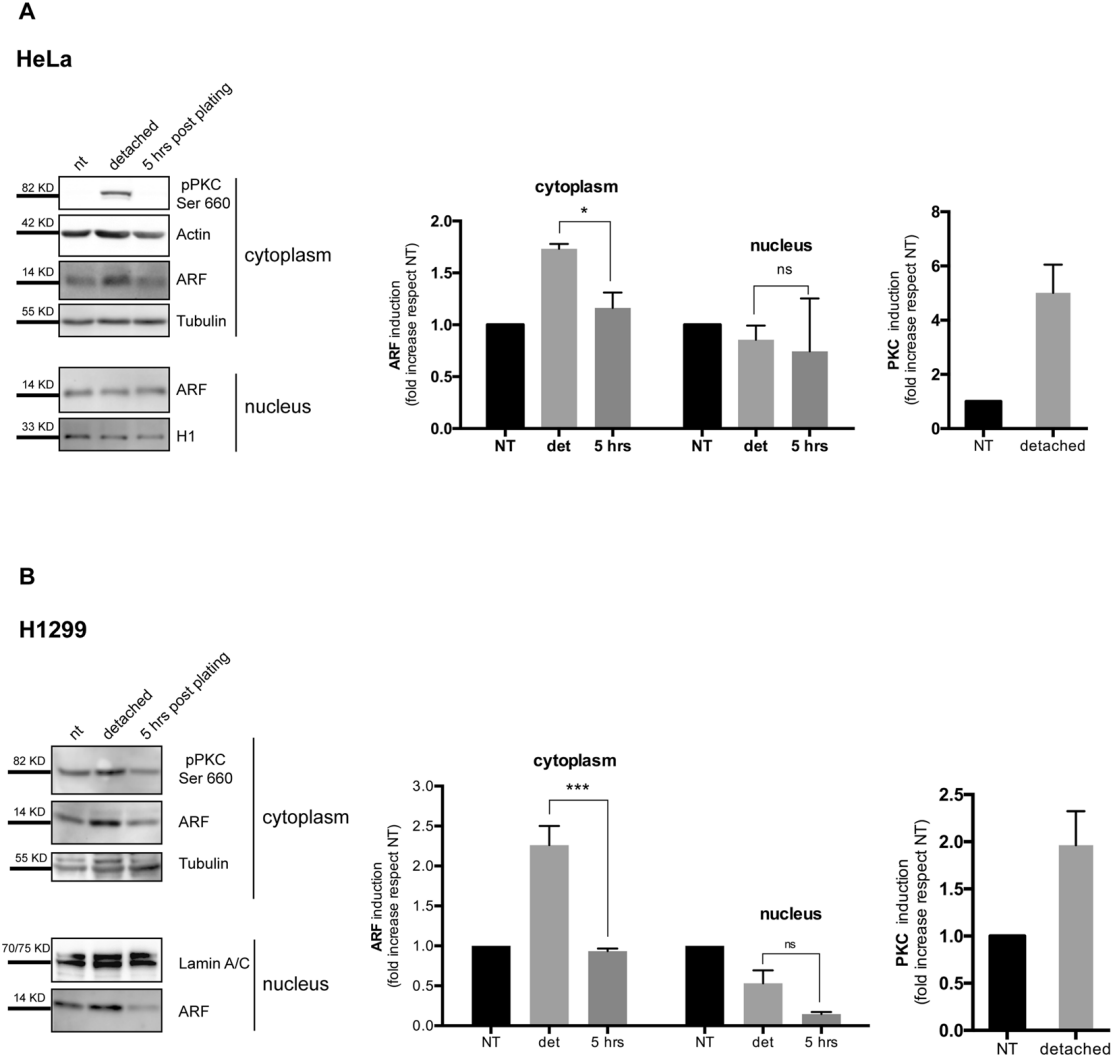
Active PKC and ARF protein levels increase upon detachment. (**A**) Fractionated cellular extracts of HeLa cells were analysed by IB with anti-pPKC Ser660, anti-ARF, anti-actin, anti-ß-tubulin (cytoplasmic loading control), and anti-histone H1 (nuclear loading control) antibodies. Representative western blots are shown. (**B**) H1299 cells were treated as in (**A**), except than lamin A/C was used as nuclear normalizer. Quantification of PKC and ARF band intensities, performed with ImageJ (See M&M for details), are expressed as fold enrichment respect to NT sample, arbitrarily set to 1. Error bars indicate S.D., n = 3. *P = 0.0124; ***P = 0.0007 by two-tailed unpaired t-test.

As the recent identified ARF role in cell spreading correlates with its cytoplasmic localization, we wondered if ARF phosphorylation could be the signal priming this new function. We first analysed if PKC activation could be detected during cytoskeleton remodelling in HeLa and H1299 cells (lung carcinoma). To this aim, we monitored levels of pPKC during cell spreading by western blot using the anti pPKC pan antibody that recognizes all the PKC isoforms phosphorylated at a carboxyl-terminal residue homologous to serine 660 of PKC β II (activated pPKC). Cytoplasmic and nuclear protein extracts were collected from untreated (nt) and detached cells, as well as from replated cells five hours after seeding, when spreading process is almost completed. As shown in Fig. 2, upon cell detachment pPKC and ARF protein levels increase in the cytoplasm (Fig. 2A and B) of both cell lines. Interestingly, ARF increase is restricted to the cytoplasmic compartment. Real time quantification of ARF mRNA levels in untreated, detached and spreading cells, shows no increase of ARF transcription in any of the tested condition in both HeLa and H1299 cells (Fig. S2) thus suggesting a post-translational mechanism involved in ARF stabilization.

**Figure 3:**
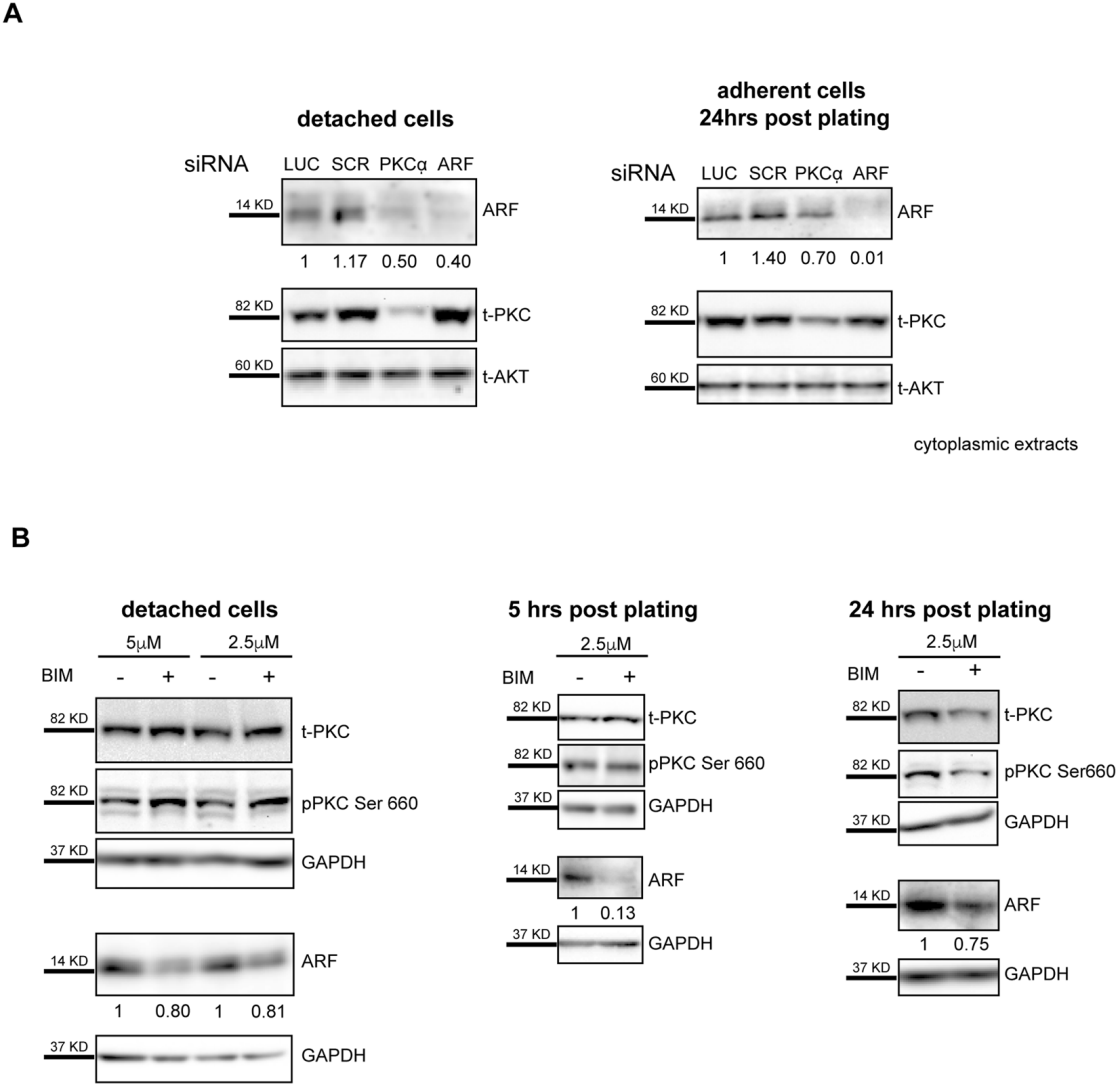
PKC activation is required for ARF induction. (**A**) Cytoplasmic protein extracts of cells collected before or 24 hours after plating analysed with anti ARF, t-PKC, and t-AKT (loading control) antibodies. ARF band intensities normalized versus AKT are shown as fold enrichment respect to siLUC. (**B**) Total extracts of HeLa cells treated with bisindolylmaleimide I (Bim) collected before, 5 hrs or 24 hrs after plating analysed with anti-ARF, pPKC, t-PKC and GAPDH (loading control) antibodies. Quantification of ARF band intensities has been performed as described.

To better analyse if the observed PKC activation is required to induce ARF increase, we knocked down PKC expression by RNA interference and analysed ARF cytoplasmic levels by western blot in HeLa cells. As control, cells were also treated with ARF specific siRNA. Control (siLuc and siSCR), PKCalpha and ARF depleted cells were collected 72hrs after transfection or subjected to trypsinization and divided in two aliquots. One was subjected to direct lysis (Fig. 3A, detached cells), while the other aliquot replated and cells collected after 24 hours. Subcellular fractionation was performed in order to analyse ARF levels in the cytoplasm. In agreement with our hypothesis, ARF levels in PKC alpha depleted cells decrease with a similar efficiency to ARF depleted cells upon detachment, remaining low until 24 hrs post plating (Figs 3A and S3). Western blot with anti-PKC antibody shows efficient silencing although small fraction of PKC molecules is resistant to silencing in line with the notion that HeLa cells also expresses epsilon and delta PKC isoforms both recognized by this antibody and not targetted by the the used siRNA. Pharmacological inhibition of PKC with bisindolylmaleimide I, an inhibitor of the catalytic subunit of PKC, confirms the requirement of catalytic active PKC in this process (Fig. 3B). Collectively these experiments show that during cytoskeleton remodelling PKC is activated resulting in increased cytoplasmic ARF protein levels.

### ARF T8D mutant rescues spreading defect of ARF depleted cells and promotes cell viability

**Figure 4:**
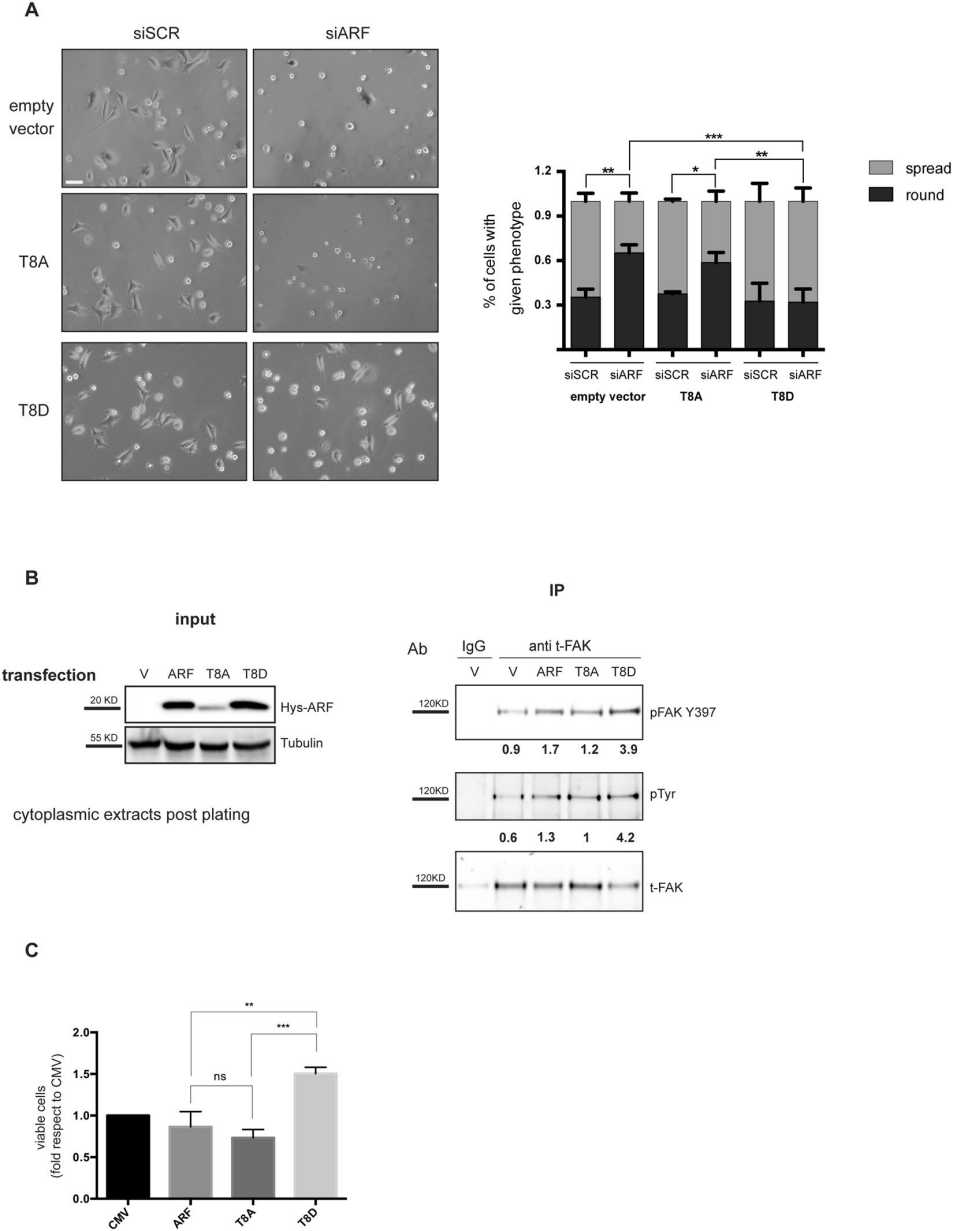
ARF T8D mutant rescues spreading defect of ARF depleted cells and promotes cell viability. (**A**) Images of HeLa cells transiently transfected as indicated, acquired by phase-contrast microscopy during cell spreading (scale bar, 30 µm) and quantification of spreading efficiency. Error bars represent SD with n = 3. P < 0.05 by two way Anova with Tukey’s multiple comparisons test. (**B**) Cytoplasmic extracts of indicated transfected cells induced to spread were immuno-precipitated with anti t-FAK antibody and analysed by western blot with anti pFAK Y397, pTyrosine, t-FAK. Panels of input are also shown. pFAK levels in the IP were normalized upon t-FAK intensities by ImageJ. Statistical analysis was performed with RM One-way ANOVA, showe a statistical trend of FAK activation with a P value < 0.01. (**C**) The plots represent the relative cell number obtained with the indicated transfection respect to empty vector set as 1. Error bars indicate S.D., n = 3. P < 0.05 by two-way ANOVA, using Tukey’s multiple comparisons test.

We previously showed that ARF silencing induces an evident morphological defect in different cell lines, including HeLa and H1299 cells. Due to the inability to properly organize the cytoskeleton structures, ARF depleted cells display a striking rounded morphology^14^. Interestingly, upon PKC downregulation by siRNA or by bisindolylmaleimide I treatment, cells showed spreading defects similarly to ARF depletion (Fig. S4). We thus explored the hypothesis that ARF phosphorylation could have a role in this mechanism. To this aim, we tested if mutant ARF proteins reintroduction is able to fulfil the function of the endogenous protein through a rescue experiment. HeLa cells were transfected with either T8A or T8D expressing vectors and empty vector as control by electroporation. Twenty-four hours later, cells were treated with scrambled or ARF-specific siRNA targeting endogenous ARF transcript (Fig. S5a). To monitor spreading process, cells were detached by trypsinization, replated and images of spreading cells collected 5 hours post plating. The ability to spread was quantified and plotted as shown in Fig. 4A. Less than 40% of ARF-depleted cells are able to properly spread both in empty vector and T8A expressing cells. In line with our hypothesis, T8D transfection is able to rescue morphology defect caused by ARF depletion in full. As shown previously, rounded phenotype caused by ARF depletion is also accompained by a decrease of FAK activation^14^. During cellular adhesion and upon integrin binding to the extra cellular matrix (ECM), FAK is activated through auto-phosphorylation of tyrosine 397 (Y397). This activation is followed by increased Src binding to FAK resulting in its massive phosphorylation onto several tyrosine residues within FAK sequence^26,27^. We thus preliminary checked if and which ARF mutant is also able to rescue FAK activation by western blot of crude extracts with anti pFAK antibodies. Results showed that in T8A expressing cells devoid of ARF expression, lower levels of both total and pFAK on tyrosine 397 are achieved, while no difference between siSCR and siARF could be detected in T8D expressing cells (Fig. S5b). The failure of T8A to induce FAK phosphorylation could be the cause of reduced spreading ability. On the other hand, this could reflect the impairment of T8A to stabilize FAK. To discriminate between these two hypotheses, we overexpressed wt and ARF mutants in HeLa cells and analysed FAK levels by immunoprecipitation followed by phospho-FAK immunodetection (both pFAKY397 and pTyr) during spreading. The experiment showed that all ARF proteins are able to positively induce FAK activation. Notably, T8D has the higher efficiency (Fig. 4B) as expressed at higher levels in the cytoplasm due to its increased stability. Our previous studies showed that FAK activation correlates with ARF ability to confer pro-proliferative properties to cells. We thus analysed if T8D mutant, in virtue of its ability to activate FAK phosphorylation, could also confer a growth advantage when expressed in cells. To this aim, HeLa cells, that endogenously express p14ARF protein and are characterized by inactivation of the p53 pathway, were transfected with plasmids encoding WT, T8A and T8D ARF mutants and empty vector (CMV) and cell growth was evaluated by comparing residual cell number 72 hrs after transfection. Comparable number of viable cells were found in both empty vector and ARF transfected samples, thus meaning that ARF expression in these cells has no effect on cell proliferation, as expected^28–30^. Similar behaviour was observed in T8A expressing cells. Interestingly, in line with our hypothesis, we constantly found an increased number of cells in T8D transfected samples (Fig. 4C). In contrast to this, similar experiment performed in H1299 cells showed no differences among wt and mutants in cell proliferation (Fig. S6).

## Discussion

Although to date the majority of studies on ARF has focused on its tumor suppressor roles, new evidences are paving the way to the hypothesis that ARF might promote survival. Here we presented evidences showing how p14ARF, upon PKC activation, is able to positively influence cell growth. The PKC family consists of at least ten serine/threonine kinases playing a central role in cell proliferation, differentiation, survival and death^31,32^. We have previously showed a novel and time-dependent ARF localization at focal adhesions upon cell spreading mirroring ARF role in cytoskeleton organization^14^. We now further characterized this aspect, showing that this function is controlled by PKC activity and can account for some of ARF pro-proliferative capabilities. While mimicking a dephosphorylated status of the protein does not alter the nuclear localization of the protein, phosphorylation of Threonine 8 is sufficient to induce an increase of ARF protein levels in the cytoplasm. More interestingly, while the no-phosphorylatable mutant appears to retain the tumor suppressor properties of the WT protein, the T8D mutant instead behaves as a constitutive active mutant conferring pro-survival properties to the cells.

The evidence that ARF functions can be thus modulated by phosphorylation events taking place on the conserved Threonine 8 leaves us to the concept that ARF role in cell survival may more realistically depends on the cellular and/or tissue type and context, thus accounting for the controversy surrounding the topic. It is interesting to underline how also the cyclin-dependent kinase inhibitor p21, in addition to the well-known growth inhibitor function, displays cancer promoting features in some cell context, as recently reported^33,34^. This behaviour, also shared by p14, suggests how the cell environment/status can act in an epistatic manner in directing tumor suppressors functions. In line with this, in H1299 cells, wt and mutants ARF proteins behave similarly as regard cell proliferation. Nevertheless, we found that T8D expression induces an increase of pFAK-Y397 phosphorylation (preliminary data not shown), thus suggesting that the effect of ARF phosphorylation on FAK activation can be disengaged from the effect on cell proliferation in these cells.

By means of pharmacological studies, protein kinase C has been implicated as a key molecule involved in cell spreading and migration, in part through interaction with beta1-integrin^35–37^. Given the context dependency of ARF role within the cell, we focused on ARF function in cell spreading in a cell context in which ARF functions as tumor suppressor are blocked, such as HeLa cells, thanks to the inactivation of the p53 pathway. Our data show that during cytoskeleton remodelling induced by detachment, both the activated form of PKC and ARF protein levels increase in the cytoplasm. We previously showed that ARF ability to favour cell spreading is accompanied by the transduction of growth signals arising from the integrin/FAK functional interaction^14^. We now added data showing that T8A can only in part rescue FAK activation, although this is not enough to allow cell spreading. This can be explained by decreased stability of the T8A mutant respect to T8D protein^15,38^. Our data depict a scenario in which during cell spreading or uncontrolled PKC activation, ARF stabilization and thus FAK activation could allow the pro-proliferative signal transduction within the cells. The effect of ARF and its mutants on cell proliferation suggested us that the T8D behaves as a constitutive active mutant. As the WT has no effect on cell proliferation this could mean that in HeLa cells something can block this ARF function. Interestingly, in H1299 cells we did not observed difference in cell growth profiles of WT and mutant proteins transfected cells. We previously demonstrated that ARF-FAK pro-proliferative axis is interrupted due undetectable levels of the Death Associated Protein Kinase (DAPK) in this cell context. DAPK is a Serine/threonine kinase playing important roles in tumor suppression and apoptosis. We previously showed that ARF expression prevents DAPK mediated anoikis. It has been shown that FAK activation can be counteracted by DAP kinase expression^39,40^ by disrupting signal transduction between integrin and FAK upon ECM interaction during spreading. This leads to the interesting hypothesis that ARF and/or T8D could be able to protect FAK by DAPK negative effect while T8A being less efficient. Interestingly, when we analysed transfected cells 24 hrs after transfection, we observed that while both controls and ARF expressing cells displayed a certain percentage of dying cells, T8D expressing cells did not (data not shown). This suggested us that T8D expression could protect cells from cell death and thus results in the observed increased cell number.

Our data led us to conceive a model in which, during cell spreading and PKC activation, p14ARF protein levels increase in the cytoplasm. It has been widely reported that PKC mediated phosphorylation regulates composition and turn-over of focal adhesions where assembly and disassembly of actin fibers take place upon different environmental inputs. In particular, cellular motility and invasivity, two of worst signals of cancer progression, are sustained by this highly dynamic phenomenon^20,41,42^. The involvement of PKC in tumor progression has thus gained recognition as potential therapeutic targets for the treatment of various malignancies. A deeper understanding of the PKC dependent ARF functions, both in physiological or pathological contexts, may provide useful information about the environmental cues that determine ARF functions as tumor suppressor or tumor promoter.

## Materials and Methods

### Cell cultures, transfection and treatments

HeLa cells were purchased from SIGMA. U2OS and H1299 were purchased from the American Type Culture Collection (ATTC) and authenticated by STR DNA Profiling Analysis. Cells were grown as described^15^ and were routinely tested for mycoplasma contamination by PCR based method and kept in culture for no more than 6 weeks after resuscitation.

ARF mutants used in this study were generated as described^15^. The cells were transfected with Lipofectamine 2000 reagent (Invitrogen) or with electroporation (Neon transfection System, Life Technologies Carlsbad, CA, USA) as described in^43^.

For RNA interference experiments, ARF siRNA (harbouring the stealth modification), that anneals in exon 1ß of p14ARF transcript, and scrambled siRNA (negative control) sequences has been reported in Vivo *et al*., 2009. PKC alfa and luciferase siRNAs are available by Qiagen (Hilden, Germany). For rescue experiment, the cells were transfected with an ARF siRNA, that anneals in the 5′UTR region of ARF endogenous transcript, as previously described in Vivo *et al*., 2017 and Kobayashi. All siRNAs were transfected using RNAiMAX reagent (Invitrogen). Rescue was performed as described in Vivo *et al*., 2017.

Treatments in this study were performed as follows: treatment with bisindolylmaleimide I (from Calbiochem): 24 hrs after plating, HeLa cells were treated either with DMSO or bisindolylmaleimide I at 5 μM and 2,5 μM final concentrations for 2 hours then were detached by trypsin to synchronize the adhesion/spreading process. An aliquot of cells was harvested and total extracts prepared for subsequent analysis as described. Another aliquot of cells was replaced in presence of bisindolylmaleimide I at 2,5 μM as final concentration and, after 5 hrs and 24 hrs post plating, the cells were harvested and total extracts prepared as described. Live phase-contrast images were acquired using a Nikon Eclipse microscope (Tokyo, Japan) with 20x objective. 5 fields were randomly selected in the plates for each experimental point and images acquired with the Image Pro Plus software (Media Cybernetics). Cell spreading was quantified as described *in Vivo et al*. 2017.

### Cell proliferation assay

4 × 10^6^ HeLa cells were transiently transfected by electroporation as described^14^. For H1299, 4 × 10^6^ cells were transiently transfected by electroporation with the indicated plasmid at 500 ng. After 72 hrs from transfection (48 hrs for H1299), the cells were counted using ScepterTM automatic cell counter (Millipore) following the manufacturer’s protocol. The data obtained were analysed using Scepter™ Software Pro (from Millipore).

Immunoprecipitation assays were performed with cytoplasmic extracts (from 5 × 10^6^ cells per sample). Subcellular fractionation was done as previously described and cytoplasmic extracts were incubated with anti-FAK C-20 antibody as described in^14^ and in^44,45^.

### WB and antibodies

Western blot (WB) analysis was performed as previously described (Vivo *et al*., 2013). Proteins were visualized by enhanced chemiluminescence (western blotting substrate Thermo Scientific). Images were acquired by ChemiDoc Imaging System equipped with a CCD camera (Bio-rad) through the Quantity One software. Representative experiments are shown for each blot. Full-length western blots are represented in supplementary information (Supplementary Figs S7, S8, S9, S10, S11, S12, S13). Band intensities were quantified by ImageJ Software (http://imageJ.nih.gov/ij/ free software, NIH), normalized respect to loading control and reported as fold enrichment respect to control sample. List of antibodies used in this study: anti-ARF C-18, anti-FAK C-20, anti-actin I-19, anti-ß-Tubulin H-235, anti-GAPDH 6C5, anti-Lamin A/C H-110, anti-Histone H1 N-16 (Santa Cruz Biotechnology, Heidelberg, Germany), anti-pFAK (Tyr397) (BD Milan, Italy; clone 14 for wb), anti-pTyr 05-321 (Upstate from Millipore, Darmstadt, Germany), anti- PKC alpha, beta and gamma M110 (Millipore, Billerica, Massachusetts, USA), anti- 6XHIS monoclonal antibody (Clontech), anti-AKT, anti-phospho PKC pan ßII Ser660; (Cell Signaling Technology, Leiden, The Netherlands).

IF and localization were performed as described in^15^, using anti His antibody to detect WT and mutant ARF proteins.

### Spreading efficiency

HeLa cells were detached by trypsinization and replated at a density of 1 × 10^5^/ml. Live phase-contrast images were acquired using a Leica DMi8 inverted microscope (Wetzlar, Germany) with 20x objective. 5 fields were randomly selected in the plates for each experimental point and images acquired with LAS X life scence (Leica). To quantify the percentage of rounded cells, for each transfection point, we counted rounded (and adherent) cells and pooled data from three to five experiments as described in^14^.

Real time experiment was performed as described^46^.

### Statistical analysis

Data presented in this work derive from experiments performed at least in triplicate (biological replicates), except when differently stated. The sample size of each experimental point is reported in the relative figure legend, as well as the specific statistical analysis performed. In all the experiments in which single cells were analysed 5 to 10 fields were randomly selected in the coverslip for each experimental point. t-test and ANOVA were performed using GraphPad Prism 5.0 software.

### Data availability

All data generated or analysed during this study are included in this published article (and its Supplementary Information files).

## Electronic supplementary material

Supplementary information
