# Supplementary material for: PKC Dependent p14ARF Phosphorylation on Threonine 8 Drives Cell Proliferation
Source: Sci Rep. 2018 May 4;8:7056. doi: 10.1038/s41598-018-25496-4 (PMC5935756; doi:10.1038/s41598-018-25496-4)

SUPPLEMENTARY INFORMATIONS

PKC DEPENDENT p14ARF PHOSPHORYLATION ON THREONINE 8 DRIVES CELL PROLIFERATION

Rosa Fontana, Daniela Guidone[∫], Felicia Sangermano[∫], Viola Calabrò, Alessandra Pollice, Girolama La Mantia and Maria Vivo

Dept. of Biology, University of Naples Federico II, Naples, Italy

FIG S1

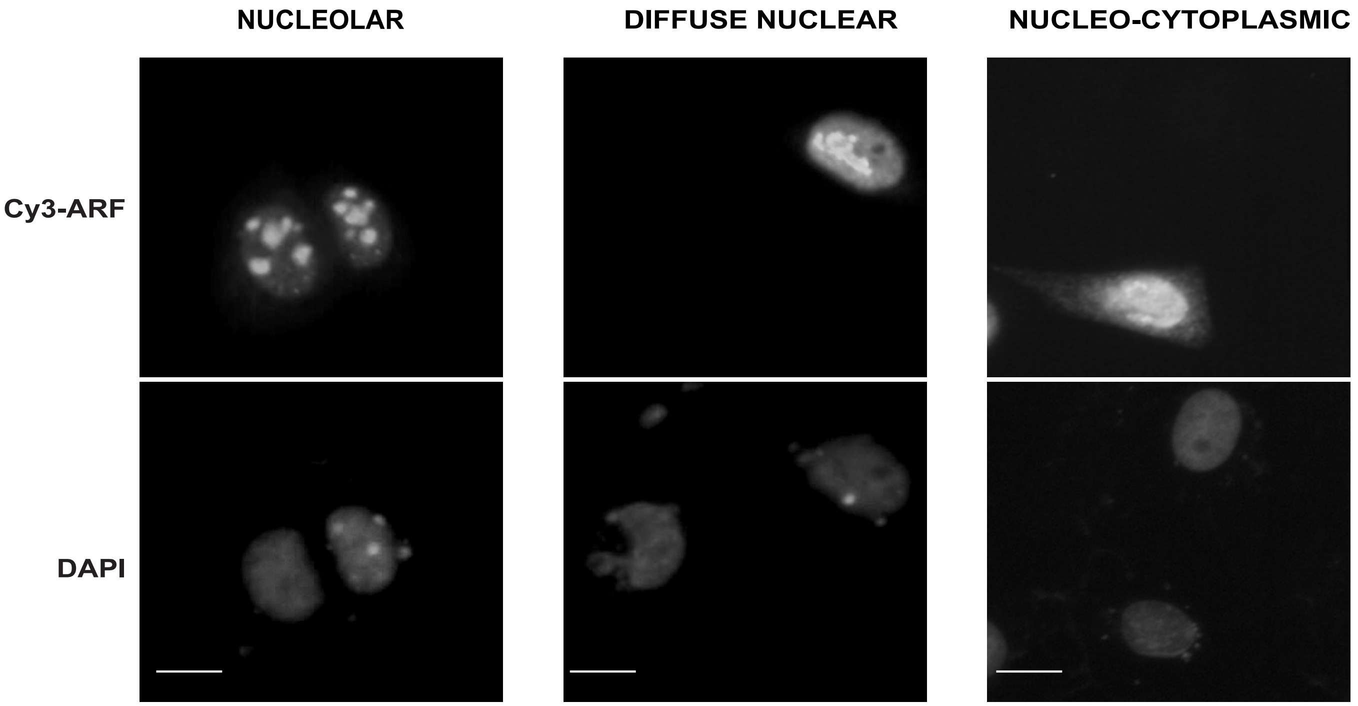

U2OS CELLS

Figure S1. Figure S1. **ARF localization analysis**. U2OS cells were transfected with p14ARF expression plasmids (wt or mutants) and visualized by IF with anti-histidine antibody. Images were taken with a Nikon fluorescent microscope. Nuclei were stained with DAPI. Examples of different ARF localization patterns displayed by ARF mutants are shown. Scale bar= 10μm The number of transfected cells showing either nuclear (nuclear+nucleolar) or nucleo-cytoplasmic localization was counted for wt and each mutant and reported in the graph in Figure 1.

Fig S2

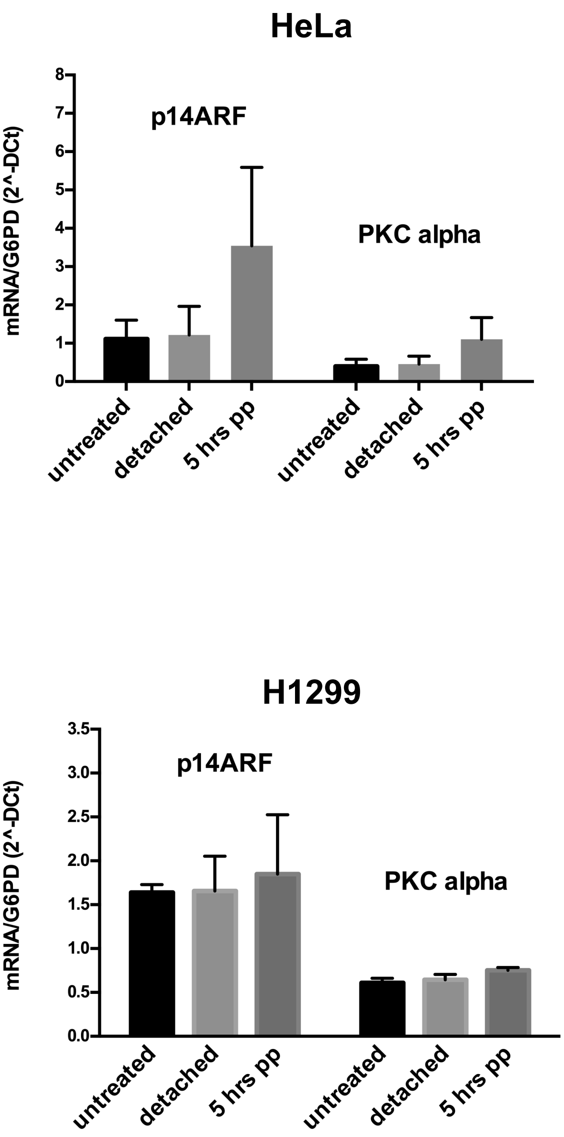

Figure S2. Quantitative reverse transcriptase (qRT)–PCR analyses of ARF and PKC alpha mRNA levels normalized on G6PD expression in HeLa and H1299 cells in different conditions. Cumulative data are expressed as a mean value ±s.d. of three independent experiments. Statistical analysis performed by 2way ANOVA show no statistical (NS) difference between samples.

Fig S3

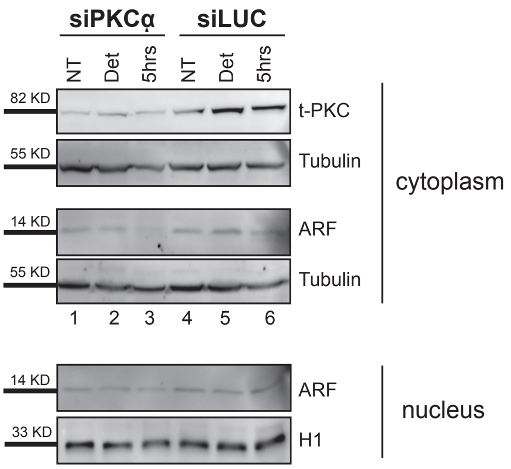

Figure S3. Effect of PKC silencing on ARF levels. Cytoplasmic and nuclear protein extracts of control and silenced cells in different conditions were treated as in Fig 3A. Western blot with anti-ARF, t-PKC, tubulin and Histone H1 (used as loading control) are shown.

Fig S4

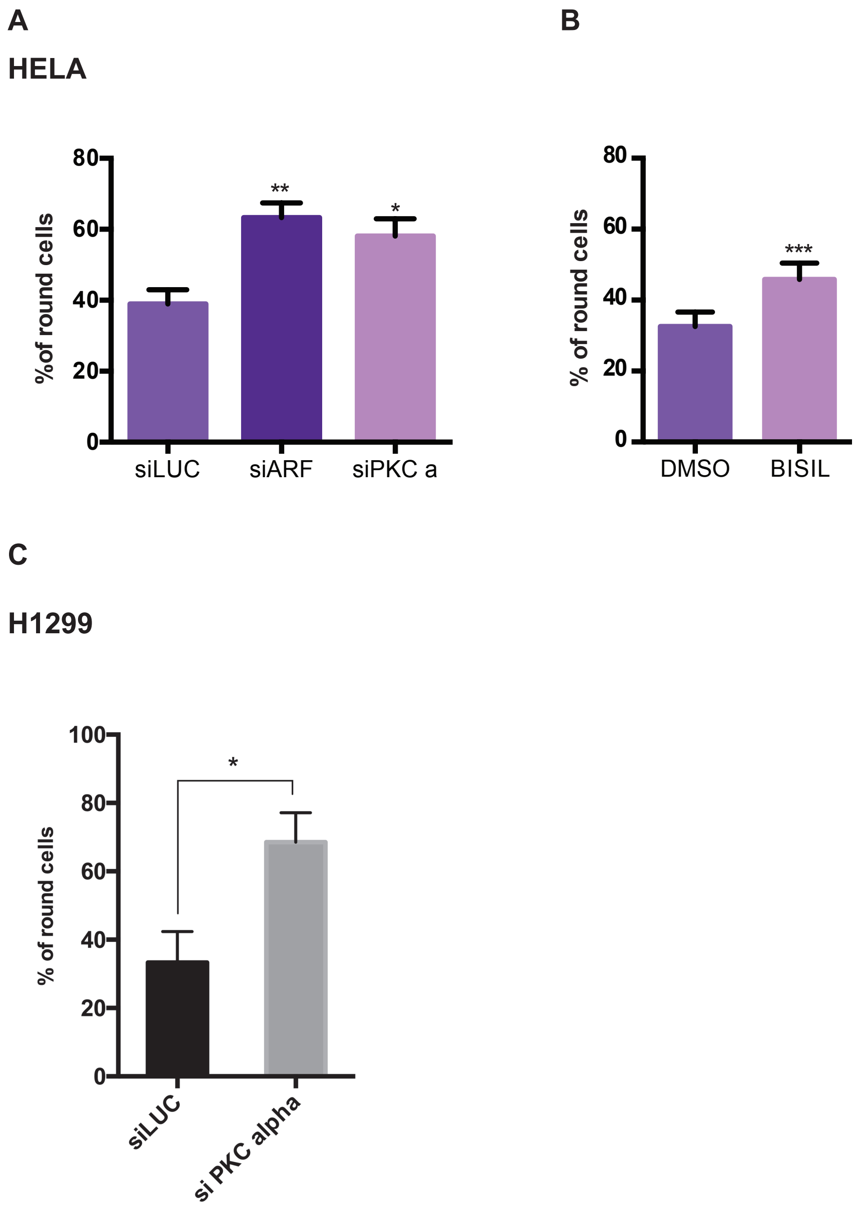

Figure S4. a) Rounded phenotype of transiently transfected HeLa cells with the indicated siRNAs for 48hrs, detached by trypsinization and replated at a density of 1X105/ml. Cumulative data are expressed as a mean value ± SEM of 4 independent experiments. Number of cells analyzed for each experiment: siSCR (200), siARF (200), siPKCalpha (200). Asterisks indicate statistically significant differences by RM One-way Anova with Tukey's correction between siARF and siPKCalpha and the control siSCR, $P < 0.05$. b) Rounded phenotype of Hela cells treated with BIM as described in Figure 4. Cumulative data are expressed as a mean value ± SEM of 5 independent experiments. Asterisks indicate statistically significant differences by two-tailed paired t-test. P=0.002. c) Rounded phenotype of H1299 cells transfected with the indicated siRNAs treated as described in a) P= 0.01

Fig S5

A

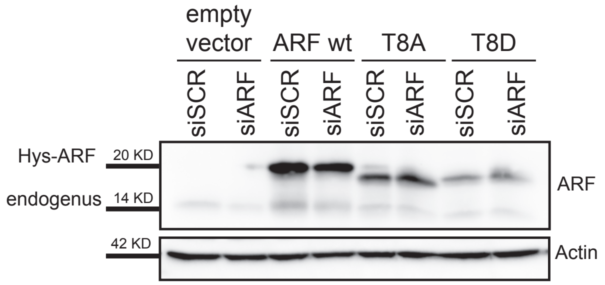

total cell extracts

B

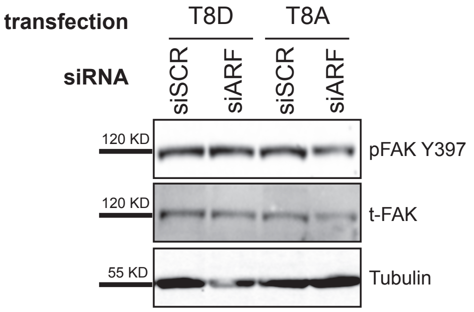

Figure S5. a) Western blot of crude extracts showing ARF mutant protein expression levels (hys tagged ARF and endogenous protein), actin is a loading control b) T8A and T8D cell extracts were analysed by IB with anti pFAK Y397, t-FAK and tubulin as control. Representative western blots are shown.

Fig S6

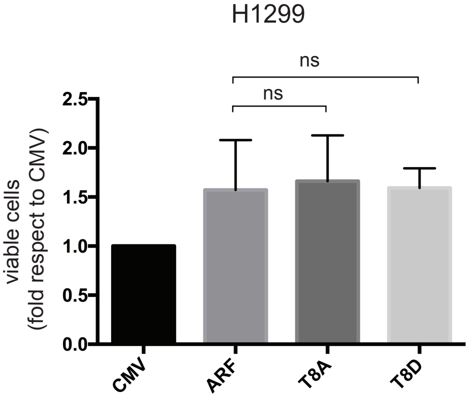

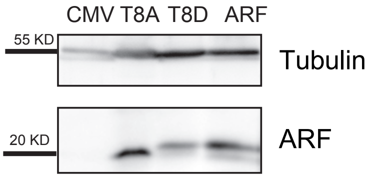

Figure S6. Equal number of H1299 cells were transfected with the indicated ARF and mutant expression vectors and empty vector as control (CMV). 48hours after transfection, the cell number in each sample was analysed with the Scepter cell count as described in M & M section. The plot represents the relative cell number obtained with the indicated plasmids respect to the empty vector set arbitrarily as 1. Error bars indicate s.d. n=3 independent experiments. Analysis of variance, performed as described before, shows non statistical (ns) differences. Western blot of total extracts showing ARF wt and mutant protein expression levels, tubulin is a loading control

Figure S7. Full-length western blots images of cropped immunoblots of Figure 2 A and B. Blue dashed squares correspond to the bands showed in the ultimate panel in the manuscript.

A

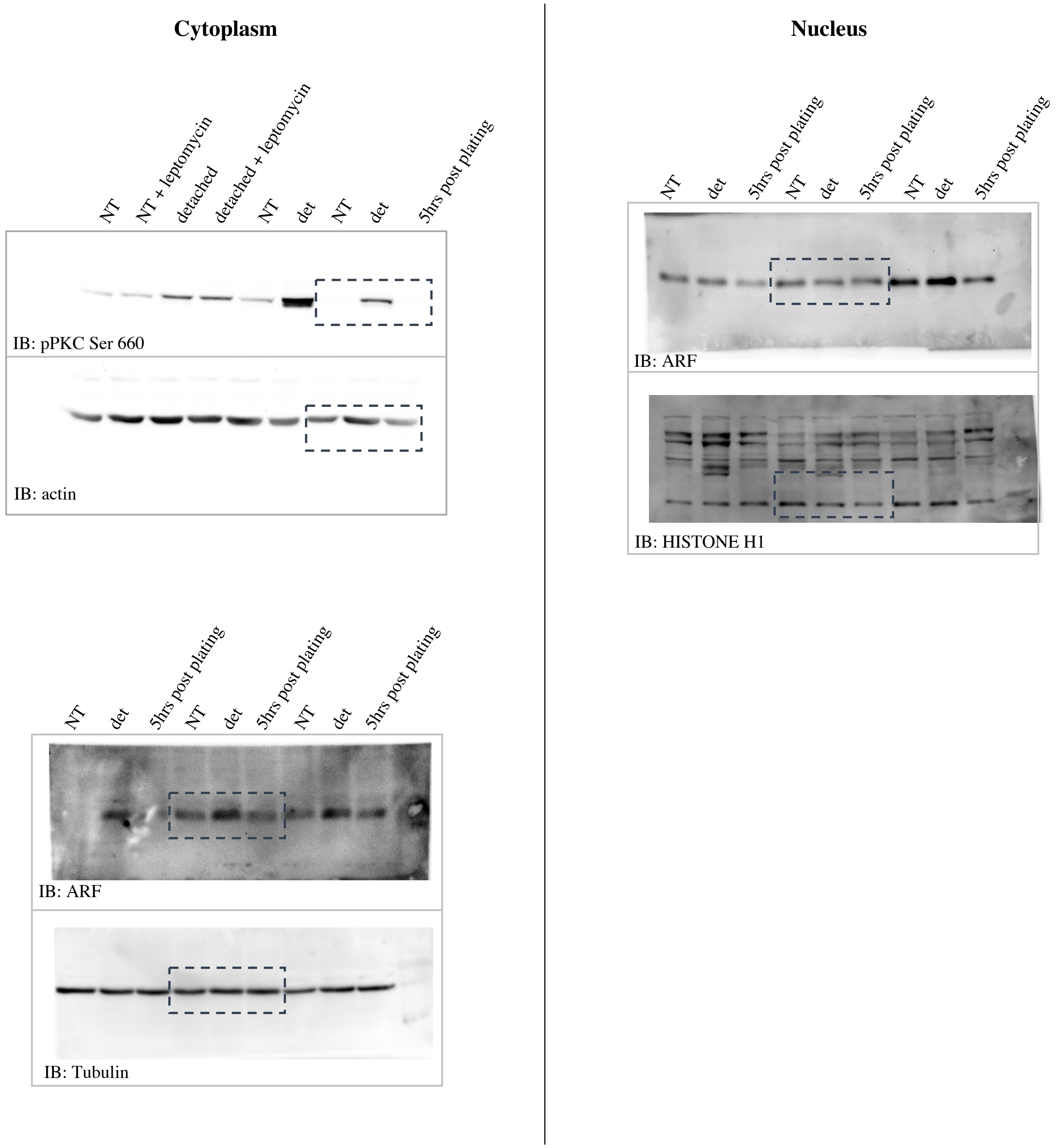

B

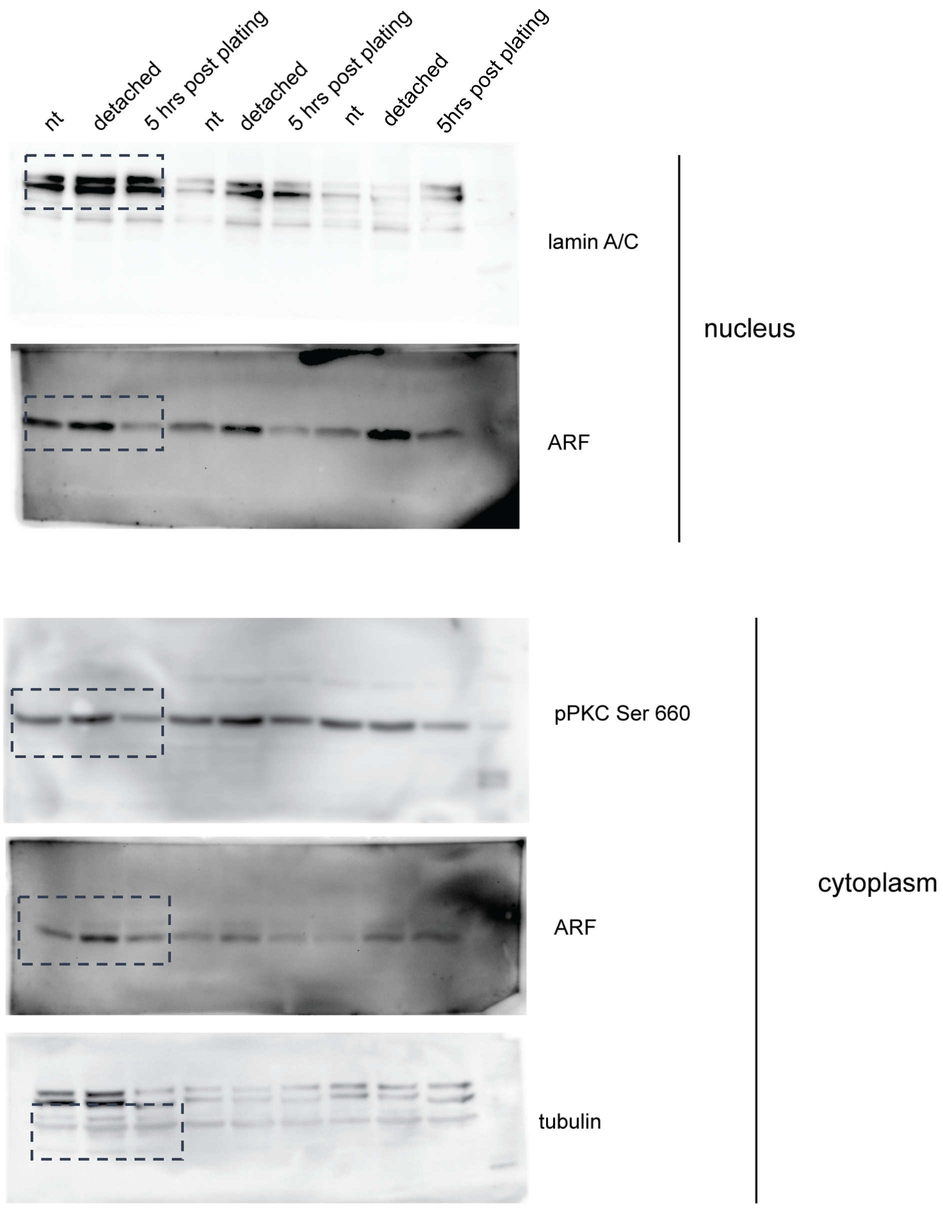

nt
detached
5 hrs post plating
nt
detached
5 hrs post plating
nt
detached
5hrs post plating
lamin A/C
ARF
nucleus
pPKC Ser 660
ARF
tubulin
cytoplasm

Figure S8. Full-length western blots images of immunoblots showing ARF cytoplasmic protein levels in HeLa cells before and after cell spreading. Blue dashed squares correspond to the bands showed in the ultimate panel in the manuscript.

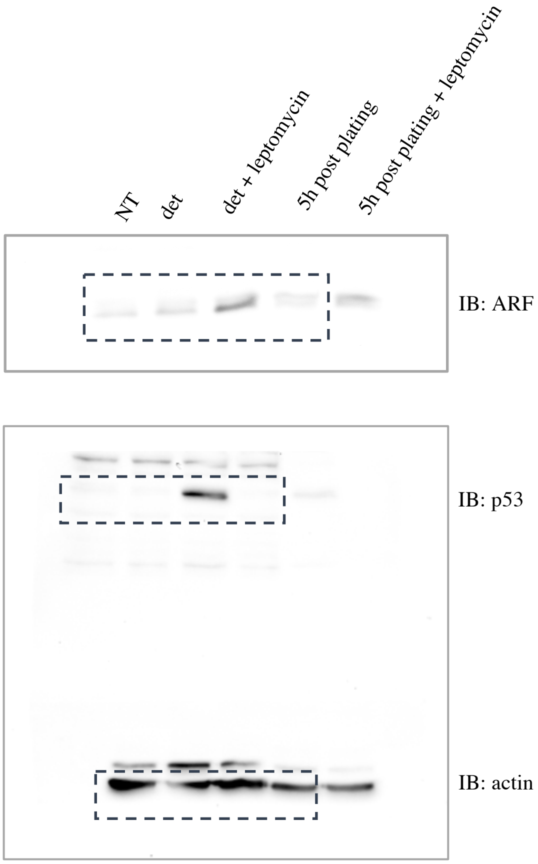

Figure S9. Full-length western blots images of cropped immunoblots of Figure 3A and B. Blue squares correspond to the bands showed in the ultimate panel in the manuscript.

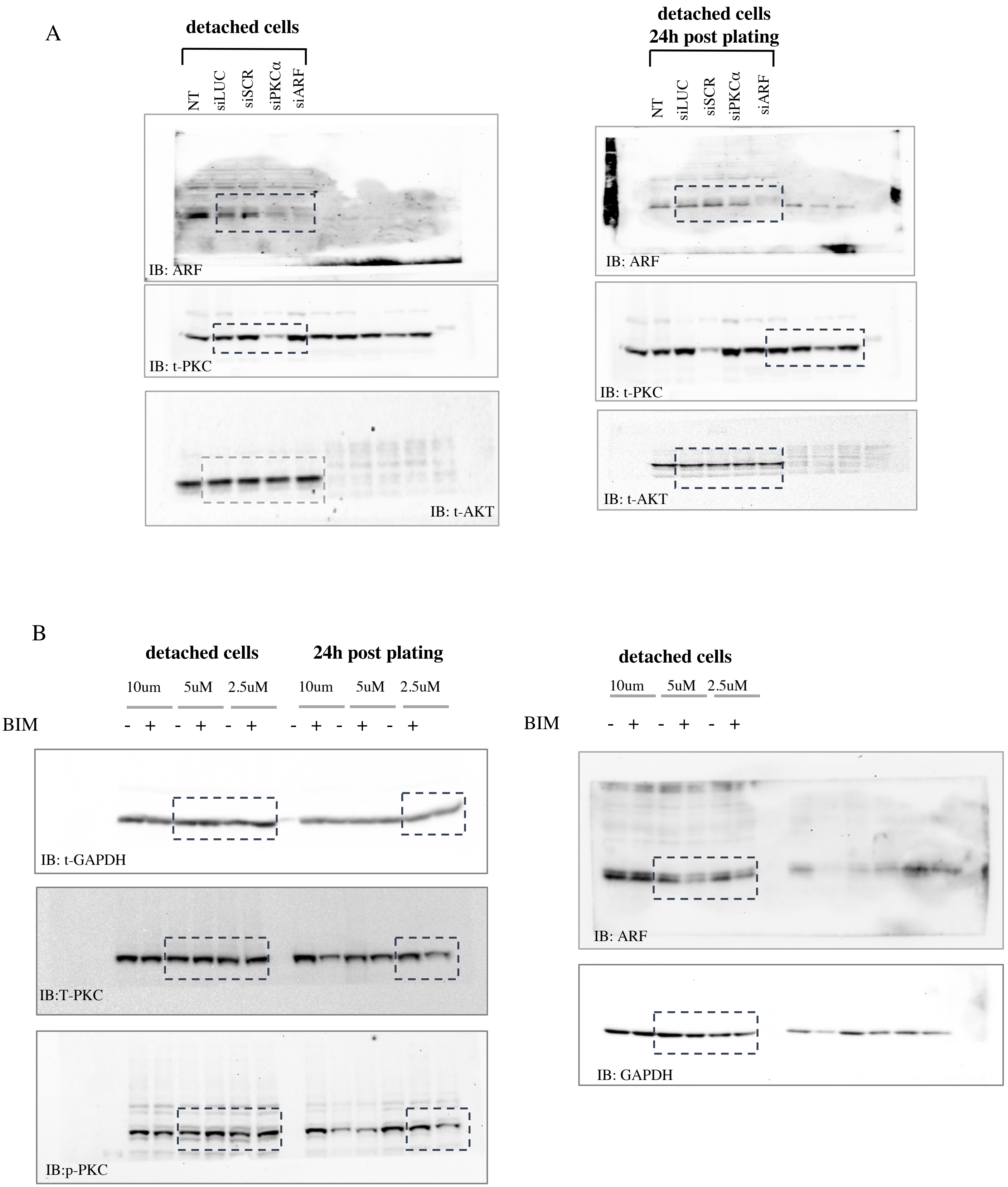

C

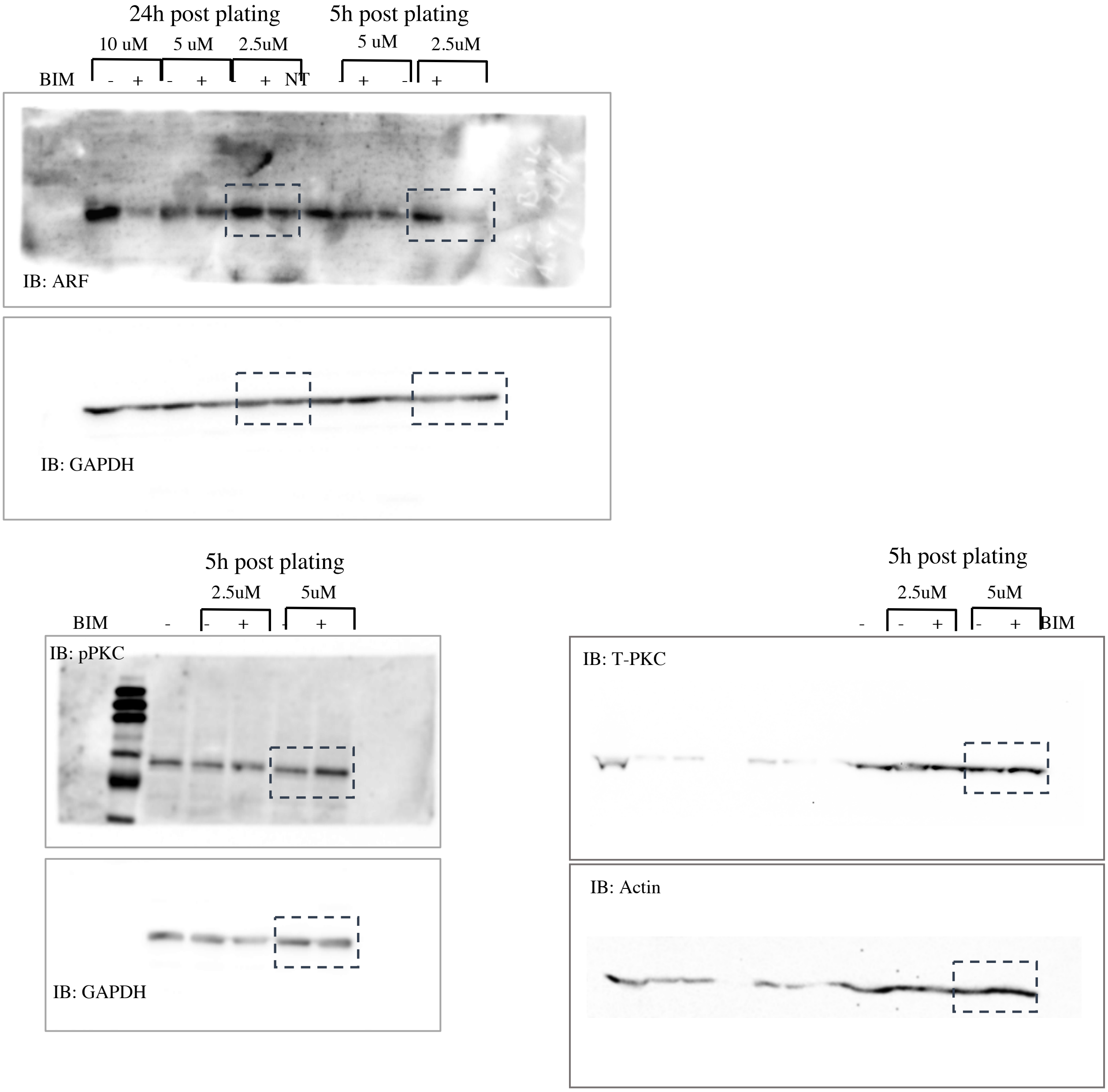
24h post plating
5h post plating
10 uM
5 uM
2.5uM
5 uM
2.5uM
BIM
-
+
-
+
+
NT
+
-
+
IB: ARF
IB: GAPDH
5h post plating
2.5uM
5uM
BIM
-
-
+
+
IB: pPKC
IB: GAPDH
5h post plating
2.5uM
5uM
-
-
+
-
+
BIM
IB: T-PKC
IB: Actin

Figure S10. Full-length western blot images of cropped immunoblots of Figure 4 B. Blue squares correspond to the bands showed in the ultimate panel within the manuscript..

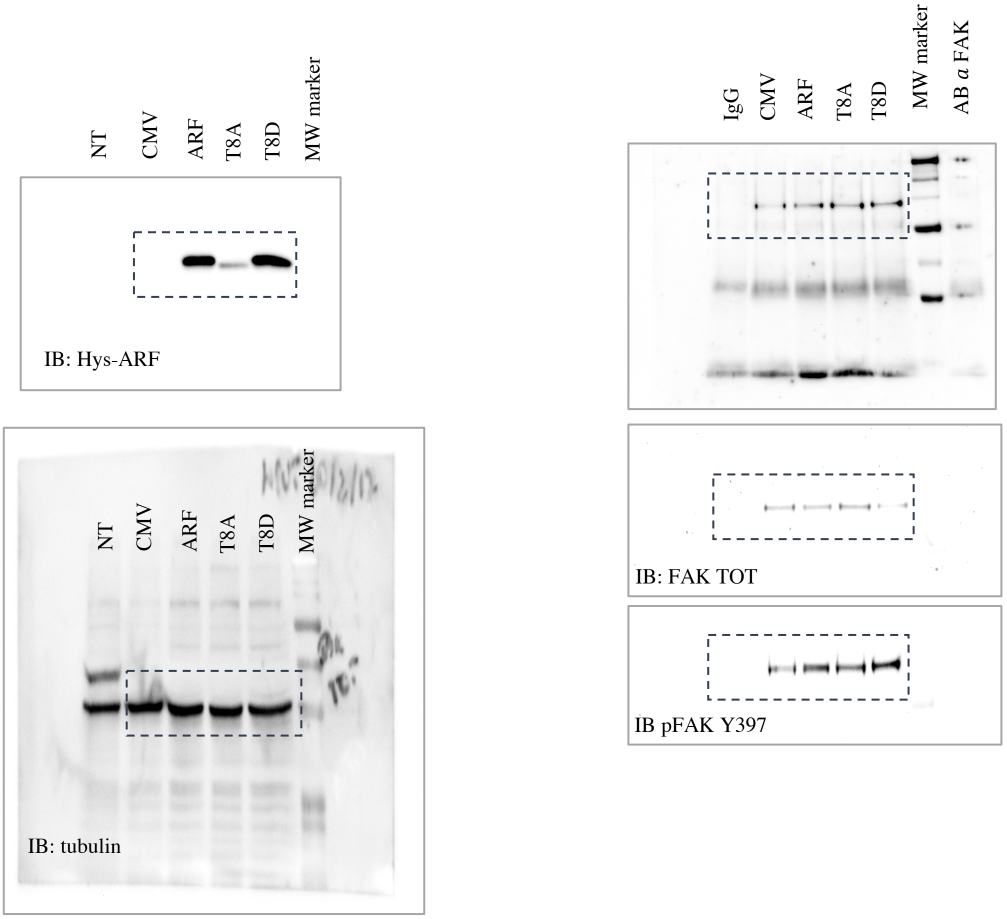

Figure S11. Full-length western blots images of cropped immunoblots of Figure S3. Blue squares correspond to the bands showed in the ultimate panel in the manuscript.

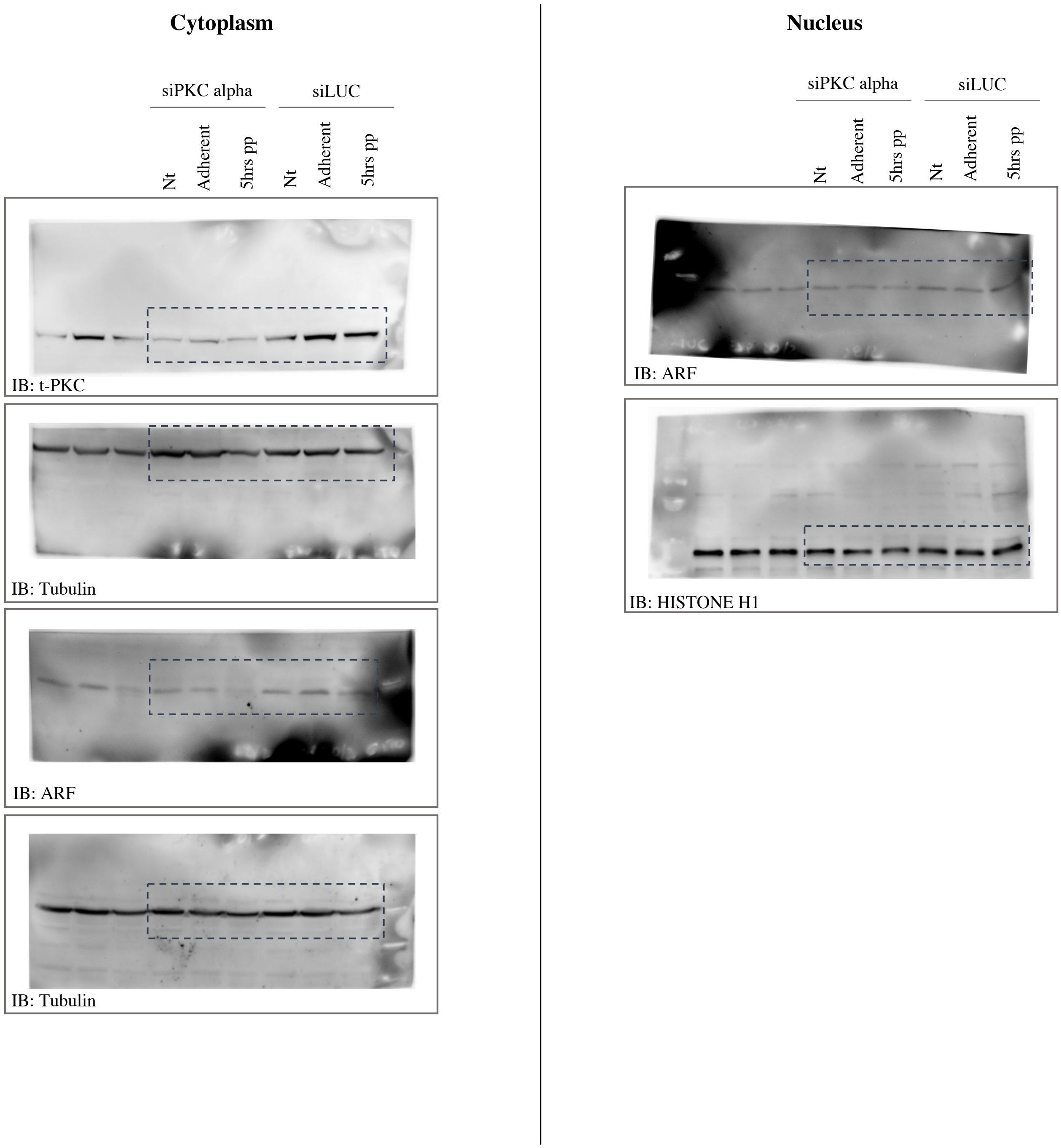

Figure S12. Full-length western blots images of cropped immunoblots of Figure S5 A and B. Blue squares correspond to the bands showed in the ultimate panel in the manuscript.

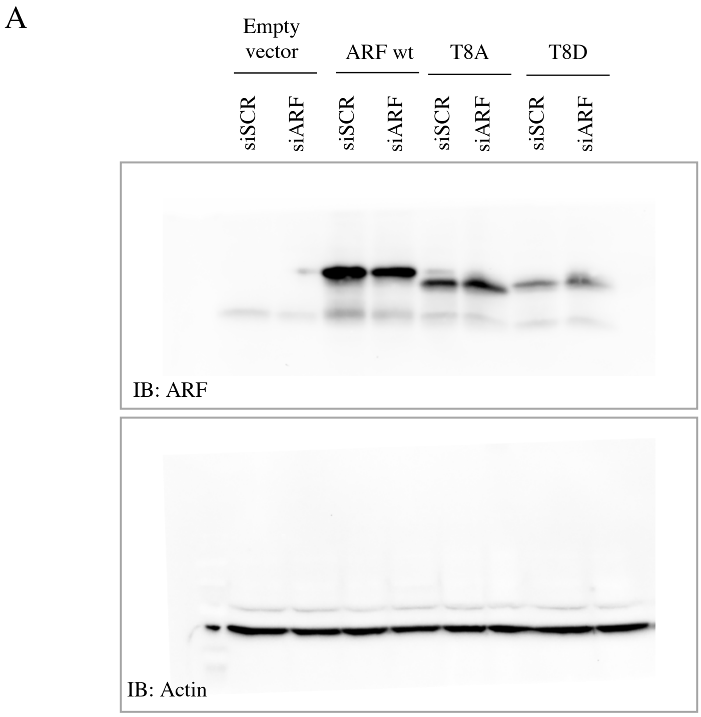

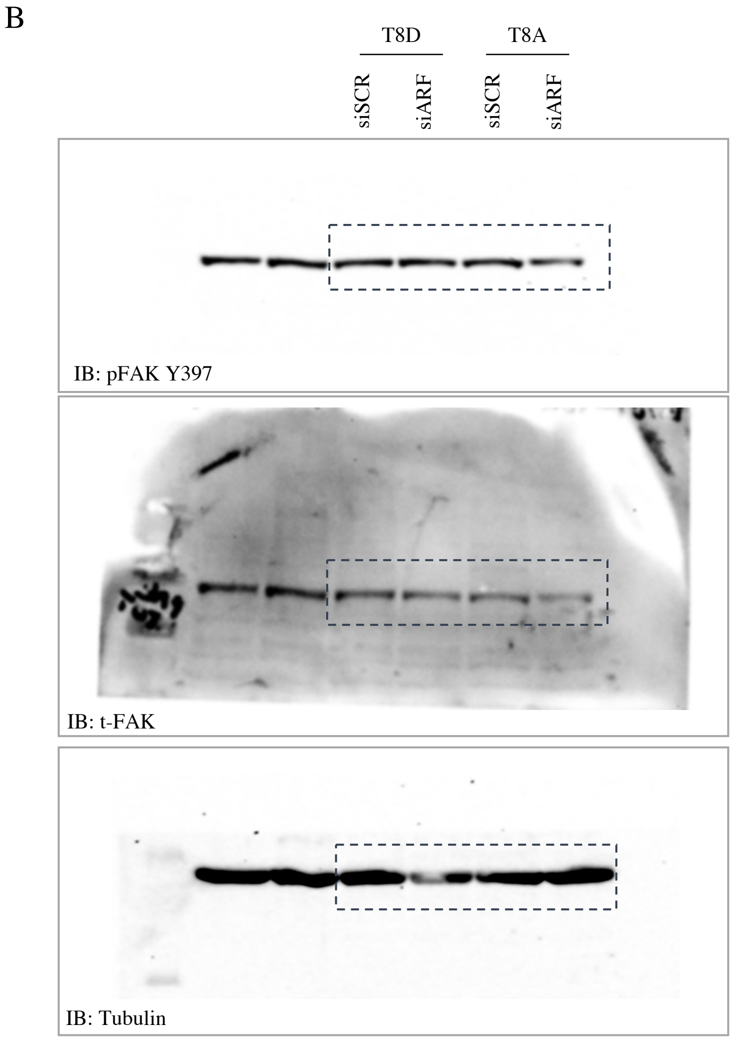

Figure S13. Full-length western blots images of cropped immunoblots of Figure S6. Blue squares correspond to the bands showed in the ultimate panel in the manuscript.

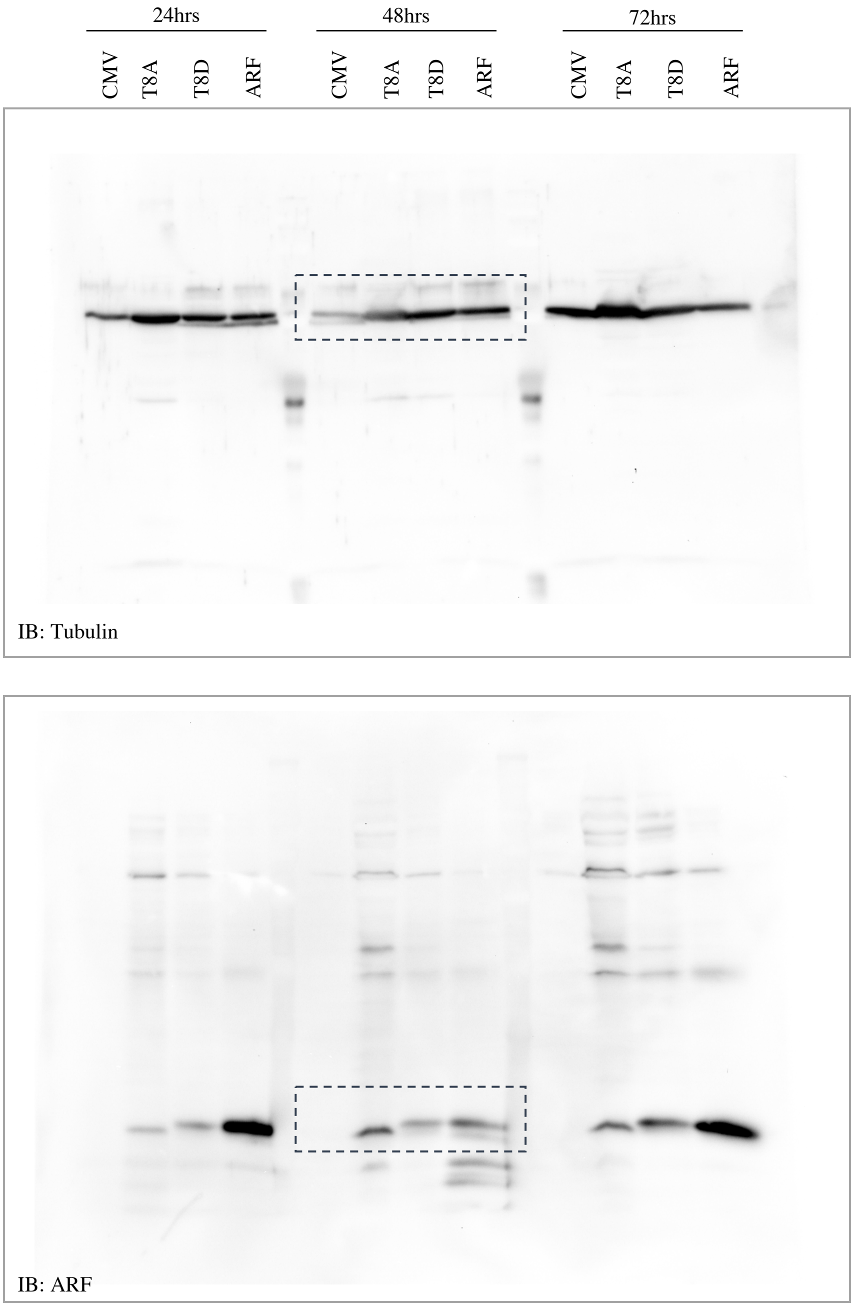

Supplement: Supplementary file 1 — Supplementary information [file 41598_2018_25496_MOESM1_ESM.pdf]
